# *Escherichia coli* O104:H4 from 2011 European Outbreak and Strain from South Korea

**DOI:** 10.3201/eid1708.110879

**Published:** 2011-09

**Authors:** Junyoung Kim, Kyunghwan Oh, Semi Jeon, Seonghak Cho, Deogyong Lee, Sahyun Hong, Seongbeom Cho, Misun Park, Dooyoung Jeon, Seonghan Kim

**Affiliations:** Author affiliations: National Institute of Health, Chncheongbuk-do, South Korea (J. Kim, K. Oh, S. Jeon, Seonghak Cho, D. Lee, S. Hong, Seongbeom Cho, M. Park, S. Kim);; Jeollanam-do Institute of Health and Environment, Gwangju, South Korea (D. Jeon)

**Keywords:** Escherichia coli, O104:H4, pulsed-field gel electrophoresis, PFGE, Shiga toxin, STEC, outbreak, Europe, South Korea, Republic of Korea, bacteria, expedited, letter

**To the Editor:** Beginning in early May 2011, an outbreak caused by Shiga toxin–producing *Escherichia coli* O104:H4 was reported in Germany and other countries in Europe. In this outbreak, the number of hemolytic uremic syndrome (HUS) cases has been unusually high (*1*). As of June 9, 2011, a total of 722 cases of HUS, 19 deaths, and 2,745 cases of enterohemorrhagic *E. coli* (EHEC) infection were reported (*2*).

A case of HUS caused by *E. coli* O104:H4 was first reported in South Korea in 2004 (*3*). Because infections caused by *E. coli* O104:H4 have been reported rarely, interest has arisen in the *E. coli* O104:H4 strain from South Korea. We characterized the *E. coli* O104:H4 strain isolated in South Korea (EC0417119) in 2004 and compared it with the *E. coli* O104:H4 strain associated with the current EHEC outbreak in Europe.

The serotype EC0417119, isolated from a patient with HUS in 2004, was reconfirmed as *E. coli* O104:H4. The strain was positive for *stx*1 and *stx*2 by PCR (*4*) but negative for *agg*R by PCR (*5*). In the antimicrobial drug susceptibility test using VITEK 2 AST-N169 test kit (bioMérieux, Marcy L’Etoile, France), the strain was resistant to ampicillin, ampicillin/sulbactam, and trimethoprim/sulfamethoxazole but susceptible to ceftriaxone, cefotaxime, nalidixic acid, and tetracycline.

We also performed pulsed-field gel electrophoresis (PFGE) for EC0417119, according to the PulseNet standard protocol (*6*), and compared its PFGE profile with that of the current outbreak strain *E. coli* O104:H4, which was obtained from the PulseNet Asia Pacific network. PFGE profiles resolved by either *XbaI* or *BlnI* did not match each other. The percentage similarity of *XbaI*- and *BlnI*-digested PFGE profiles of the 2 isolates was 75% and 66.7%, respectively, as shown in the Figure.

**Figure:**
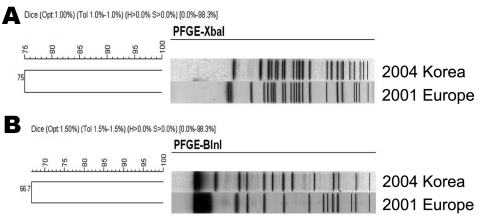
Clustering of A) *XbaI-* and B) *BlnI-*digested DNA fragments by pulsed-field gel electrophoresis (PFGE) for *Escherichia coli* O104:H4 2011 outbreak strain in Europe and isolate obtained in South Korea in 2004.

Infections with the EHEC O104 strain were reported several times worldwide. In Europe, such occurrence was rare, and before the current outbreak, the EHEC O104:H4 strain was documented only once in South Korea. For this reason, it was logical to examine the possible relatedness of the EC0417119 strain and the strain causing the current outbreak. However, the EC0417119 strain has many different characteristics compared with the current outbreak strain: not possessing enteroaggregative *E. coli* determinant, not producing extended-spectrum β-lactamases, and not showing indistinguishable PFGE patterns. In conclusion, there is no evidence that the *E. coli* O104:H4 strain isolated in South Korea in 2004 is related to the strain that has a caused the massive and unprecedented EHEC outbreak in Europe.
